# The role of community-based approaches in achieving universal health coverage: addressing the Nigerian narrative

**DOI:** 10.1097/MS9.0000000000000443

**Published:** 2023-04-03

**Authors:** Fortune B. Effiong, Chiemela P. Ogbonna, Prosper I. Agughalam, Miracle O. Okwukwu, Innocent C. Dike, Emmanuel E. Elebesunu, Olivier Uwishema

**Affiliations:** a Directorate of Research, TORASIF, Calabar; b Department of Clinical Chemistry and Immunology, Faculty of Medical Laboratory Science, University of Calabar, Calabar; c African Community for Systematic Reviews and Meta-Analyses (ACSRM), Kigali; d Department of Medical Laboratory Science, Abia State University, Okigwe; e Department of Medicine and Surgery, College of Health Sciences, University of Port-Harcourt, Port Harcourt; f Department of Medical Laboratory Science, Nnamdi Azikiwe University, Awka; g Department of Medicine and Surgery, University of Ibadan, Ibadan; h Department of Medical Laboratory Sciences, University of Nigeria, Enugu, Nigeria; i Oli Health Magazine Organization, Research and Education, Kigali, Rwanda

**Keywords:** community health, Nigeria, Africa, primary health care, universal health coverage

## Abstract

Universal health coverage (UHC) is aimed at ensuring that individuals and communities have affordable access to essential health care services without facing financial hardship. Achieving UHC and the third sustainable development goal of the United Nations requires that health systems transition from a vertical, top-down, curative approach toward one that puts people at the core of health care services, such as community-centered health interventions. Nigeria operates a decentralized health care system with the least focus on primary health care, making access to quality, and affordable health care for several citizens a challenge as the major percentage of the Nigerian population relies on primary health care services. The limited number of health care workers, the poor economic state, the inadequate health financing structures and high illiteracy rates have led to challenges such as low health service availability, hesitancy to utilize health interventions, high out-of-pocket expenditure rates, and health misinformation. These can be effectively tackled at the community level by revamping primary health care services, adequate and sustainable health financing, establishing Ward Development Committees, and the involvement of community stakeholders in health policy implementation. Employing such community-based approaches will ensure continuous progress of the Nigerian health care system toward UHC.

## Introduction

Highlights

- Universal health coverage is aimed at ensuring that individuals and communities have affordable access to essential health care services without facing financial hardship.
- It encompasses the full range of basic, high-quality health services from prevention to recovery, rehabilitation, and palliative care for people of all ages.
- Achieving universal health coverage and the third sustainable development goal of the United Nations requires that health systems transit from a vertical, top-down, curative approach toward one that puts people at the core of health care services, such as community-centered health interventions.

Universal health coverage (UHC) aims at ensuring that individuals and families have access to the health care needed without facing financial hardship^1^. It encompasses the full range of basic, high-quality health services from prevention to recovery, rehabilitation, and palliative care for people of all ages^1^.

Nigeria is the most populous nation in Sub-Saharan Africa, and the National Population Council (2019) predicts that by 2050, its population would have doubled to 400 million^2^. Nigeria ranks 145 out of 195 nations in the Lancet evaluation of health systems’ effectiveness based on access to and quality of health care^3^. Nigeria likewise performs poorly in the delivery of services for UHC, according to the World Bank’s Universal Health Coverage Service Coverage Index^4^. Health care in the country is a concurrent responsibility of the three tiers of government. Only a few states with political commitments have been able to make progress in improving health outcomes because the state government holds significant political power and is primarily responsible for the health services provided there^5^. The local government has little resources and capacity, which makes it difficult for it to improve primary health care (PHC) services. In general, the Nigerian government’s inability to improve health care in the nation originates from weak leadership and accountability structures, inadequate qualified health care workers, and a lack of transparency procedures inside the system^6^.

Achieving UHC and the third sustainable development goal (SDG 3) of the United Nations in Nigeria requires the health system to transit from a vertical, top-down, curative approach toward one that puts people at the core of health care services^7^. When community members are actively engaged, it creates a workforce capable of remaining receptive, forming relationships, managing processes and providing qualitative and quantitative data that can help health services become more integrated, coordinated, adoptive, and responsive^7^. Also, when community members are engaged in health programs, it gives a sense of ownership, bringing health to their doorstep which helps in proper integration of health interventions. Access to health services leads to better health and good health is interlinked with economic development and poverty reduction; therefore, good health directly affects all other SDGs. Countries such as Malawi, Ethiopia, Rwanda, and India have achieved remarkable success and have experienced improved health outcomes by expanding their community health programs^8^. Nigeria faces challenges that delay progress toward the attainment of the national government’s declared goal of UHC. An approach that is people focused is indispensable for advancing UHC in Nigeria. This article explores four ways in which community-centered interventions can be used in advancing UHC in Nigeria (Table 1).

**Table 1:** Problems, solutions, and community approaches for universal health coverage.

Problem	Solution	Community approach
Health service availability	Revamping of Primary Healthcare Centres	Community Health Workers
Health service affordability/out-of-pocket spending	Sustainable health financing system and political will	Community-Based Health Insurance Scheme
Health service uptake	Tackling health misinformation through awareness campaigns	Ward Development Committees
Health service acceptance	Involvement of community stakeholders in health policy implementation processes	Community Stakeholders

### Tackling health service availability: the role of community health workers and PHC

During the Alma-Ata declaration of 1978, PHC was defined as essential health care universally available to individuals at both the community and national level, taking into consideration the coat of care and keeping it at the level easily achievable by every individual, at every stage of their development^9^. Introduction and implementation of good PHC in Nigeria will curb the spread of diseases and infections, bring down the level of infant mortality and maternal mortality, arrest pandemics, minimize out-of-pocket spending and enhance good and quality health care^10^. In Nigeria and many other Sub-Saharan countries, the limited number of health care workers is the leading cause of poor health care at the PHC level^11^. The concept of using community people to provide basic health services has been identified as one strategy to address the growing dearth of health workers. Community Health Workers (CHWs) can make significant progress in establishing PHC by acting as trusted liaisons between communities and the formal health care system. They can better communicate with and gain trust from their patients; can create culturally relevant and easily accessible health materials and information. Moreover, they know how their neighbors live and think and can assist in the adaptation of the health care system to better meet the needs of their population^12^, thus, acting as cost-effective extensions of the health system. With these capacities, CHWs in Primary Healthcare Centres can successfully engage large numbers of community members in raising knowledge and reducing barriers to quality health care quality^13^.

PHC is essential to enhancing national health systems and must be given top priority. A more effective network of PHC facilities can improve the delivery of public health (preventive and promotive) services, the availability of free diagnostics and medications, and the efficient enforcement of health regulations, ensuring that all populations have access to high-quality health care that is both available and accessible^8^. There is a need for stakeholders to launch appropriate policy reforms and allot the required funds that will sustain Primary Healthcare Centres and empower CHWs to ensure the availability of health services in Nigerian communities.

### Tackling out-of-pocket spending: the role of community-based health insurance schemes

The functioning of health systems and the achievement of UHC are dependent on health financing. Health care systems should have an operational mechanism in place to gather and pool resources to make strategic purchases of necessary supplies of health services to those in need^14^. Putting into context Nigeria’s poor economic state, the need for a contributive financing that can lift the burden from the government cannot be overemphasized. Health financing structures in Nigeria are characterized by low government spending, insurance schemes that are not well developed, high out-of-pocket spending and a high reliance on external sources^15^. Nigeria’s reliance on out-of-pocket spending prevents millions of people from accessing health care and has projected millions into poverty^16^. Therefore, there is a growing need to significantly reduce out-of-pocket spending and tap into domestic resources to make progress toward achieving UHC. Capital derived from domestic resources will be beneficial, more predictable, facilitate better planning, and implementation giving an edge over donor funds. Furthermore, it will promote a greater sense of control and responsibility at various stages of the health care system^14^. Enrollment in public health insurance has been shown to improve the physical and mental health status of beneficiaries, decrease the prevalence of acute or chronic diseases and increase the likelihood of good health and satisfaction^17^. The various types of health insurance schemes in Nigeria include community-based health insurance (CBHI), state-based health insurance and National Health Insurance Schemes (NHISs)^18^.

The Nigerian NHIS was formally launched on 6 June 2005, but since then, only about 5% of Nigeria’s population is covered by this insurance scheme, most of which are workers directly under the federal government^19^. Thus, a huge percentage of the population in Nigeria still bears the brunt of directly paying for their health services. In order to improve coverage, the Federal Government of Nigeria included a regulated CBHI model and State Supported Health Insurance Programme within its NHIS^19^ They are mechanisms within the NHIS through which the informally employed and rural members of the population can obtain health care coverage.

Community health insurance programs are one way to pool community resources to share in the financing of local health services^20^. With the existing Nigerian health care system, the rate of out-of-pocket medical expenses is still horrendously high. In order to promote fairness in access to health care services and effective financial risk protection, recommended approaches must be adopted immediately. Although CBHI may not be appropriate in every circumstance, it can be crucial to cutting out-of-pocket spending and making health services affordable to community members.

### Tackling health misinformation: the role of Ward Health Development Committees

Misinformation can foster an atmosphere of panic and fear. Health misinformation can be fueled by religious/traditional beliefs, political propagandas, and illiteracy^21^. Health misinformation can cause low uptake of health services even when these services are available, accessible, and affordable. For instance, Islamic clerics’ anti-polio propaganda, seen as a nefarious plot by Western governments to reduce Muslim women’s fertility decreased uptake of the polio vaccine even when it was available, accessible, and affordable.^22^ Also, during the early phase of the coronavirus disease 2019 pandemic, there were rumors about coronavirus disease 2019 testing being a plot by the Western and Chinese government to introduce the virus to the African population that were ‘immune’ to the virus. These rumors delayed diagnosis and contributed to the community transmission observed in the early phase of the pandemic^23^. Hence, there is a need to provide clear and up-to-date information to the public at all stages of a public health response effort.

Strengthening the Ward Development Committee (WDC) or Ward Health Development Committee (WHDC) in every Nigerian committee is one approach to spot misinformation and track it before it goes viral. The WDC is a National Primary Health Care Development Agency initiative that aims to strengthen local communities’ capacity to disseminate health information while taking into context the differential demographic peculiarities of the communities^24^. It gives the local communities information that helps them make informed choices, change risky health practices, and ensures greater acceptance of novel health interventions, as well as their use and sustainability^24^. The platform that the WHDC provides is important because it provides feedback for the rumors that are circulating in the community around a particular health issue. Health educators are able to learn about these rumors and provide appropriate measures/suggestions for them to be debunked. Stakeholders should strengthen the WDC in every community by providing the needed funding and infrastructure; especially vehicle infrastructure and instructional materials including posters for both indoors and outdoor publicity and enlightenment campaigns. This will help address the growing problem of misinformation, and increase uptake of health services by community members.

### Multisectoral and multilateral collaboration with community stakeholders

Treading the path toward UHC requires the participation and support of diverse disciplines, professions, and institutions beyond the health sector. Understanding how to collaborate with diverse community stakeholders’ groups in a way that respects and builds on their culture, knowledge and perspectives is critical in developing more long-term community owned solutions. Especially because it is no longer about how much knowledge health practitioners have, but rather how they can apply their knowledge, collaborating with others that makes a difference.

The complexity of health problems in today’s globalized world is challenging the decentralized and usually isolated approaches of contemporary medicine and public health^25^ as a single health issue may be influenced by interrelated social, environmental and economic factors that can best be addressed with a holistic multisectoral approach. A multisectoral strategy entails deliberate collaboration among diverse community stakeholders’ groups (e.g. government, civil society, and private sector) and sectors (e.g. health, environment, and economy) in order to achieve a common policy goal^26^. When several sectors get involved, partners can pool their knowledge, expertise, and resources to achieve a common goal of improved outcome.

A key strategy for incorporating people-centered health systems is to engage and inspire these community stakeholders. Their potential for promoting health should be encouraged. Some of the community members can be elected as part of the planning, management, and monitoring bodies of health programs. Private sector should work together to raise the necessary resources and harness technology in order to ensure that service delivery is sustained and no one is left behind^27^. A conducive legal, policy, and regulatory environment is required for increased involvement with the private sector, as well as continuous dialog between the private and public sector. As a result, the government should identify relevant areas for partnering and contracting the private sector in order to broaden service coverage and establish adequate mechanisms to ensure shared openness and accountability^27^. In India, for example, community and civil society engagement was promoted for programmes focused on HIV/AIDS, tuberculosis, polio, and immunization^28^–^30^. Community members and stakeholders were brought together by the community action for health model to evaluate their public health systems and advocate for improvements. Successive assessments and evaluations has shown increased performance of several service delivery parameters^28^,^29^. The importance of multisectoral strategy at the community level in achieving UHC and SDGs in Nigeria cannot be overemphasized, as it is essential for breaking down silos, avoiding community opposition and increasing health uptake, all of which can help achieve UHC and SDGs.

## Conclusion

Strengthening PHC workers will solve the problem of health service availability. Building a sustainable health financing system will solve the problem of affordability. Even when health interventions are available and affordable, misinformation can cause a negative perception of the intervention. Strengthening WDC is a good way of tackling misinformation at the community level and increasing acceptance and uptake of health interventions. Lastly, it is important to collaborate with multistakeholders at the community level in order to increase acceptance, reduce opposition and achieve a common policy goal. These approaches will keep Nigeria in the race toward UHC.

## Ethical approval

Not applicable.

## Consent for publication

Not applicable.

## Sources of funding

None.

## Author contribution

F.B.E.: conceptualization, writing – original draft. F.B.E., C.P.O., P.I.A., O.M.O., I.C.D., E.E.E., O.U.: writing, review and editing, resources. F.B.E., C.P.O., O.U.: supervision, validation, review, and editing. All co-authors have read and approved the final manuscript.

## Conflicts of interest disclosure

No conflicts of interest declared.

## Research registration unique identifying number (UIN)

None.

## Guarantor

Fortune Benjamin Effiong.

## Data availability statement

Not applicable.

## Provenance and peer review

Not commissioned, externally peer reviewed.
